# Parkinsonian Rigidity Depends on the Velocity of Passive Joint Movement

**DOI:** 10.1155/2015/961790

**Published:** 2015-12-16

**Authors:** Takuyuki Endo, Naoya Yoshikawa, Harutoshi Fujimura, Saburo Sakoda

**Affiliations:** ^1^Department of Neurology, Toneyama National Hospital, 5-1-1 Toneyama, Toyonaka, Osaka 560-8552, Japan; ^2^Graduate School of Engineering Science, Osaka University, 1-3 Machikaneyama, Toyonaka, Osaka 560-8531, Japan

## Abstract

*Background.* It has been long believed that Parkinsonian rigidity is not velocity-dependent based on the neurological examination. However, this has not been verified scientifically.* Methods.* The elbow joints of 20 Parkinson's disease patients were passively flexed and extended, and two characteristic values, the elastic coefficient (elasticity) and the difference in bias (difference in torque measurements for extension and flexion), were identified from a plot of the angle and torque characteristics. Flexion and extension were done at two different velocities, 60°/s and 120°/s, and a statistical analysis was performed to determine whether the changes in these characteristic values were velocity-dependent.* Results.* The elastic coefficient was not velocity-dependent, but the difference in bias increased in a velocity-dependent manner (*P* = 0.0017).* Conclusions.* The features of rigidity may differ from the conventional definition, which states that they are not dependent on the velocity of joint movement.

## 1. Introduction

Rigidity and spasticity are two well-known abnormalities of muscle tone. Rigidity is a major characteristic of Parkinson's disease (PD), and it has been distinguished from spasticity in that the resistance of a joint is typically described as constant regardless of the joint angle and is not dependent on the velocity of the movement [1]. (We regard “velocity” as “angular velocity” in this report.) However, such a definition depends on the subjective method of neurological examination, and it should be confirmed by a scientific measurement system. With respect to rigidity in PD patients, Lee et al. quantified the velocity-dependent features of muscle tone using a torque meter when the elbow was flexed at a constant speed, and they showed velocity dependence [2]. However, they did not show that the characteristic values used in that study were correlated with rigidity in clinical assessments. The pathophysiology of Parkinsonian rigidity has been investigated using electrophysiological technique for a long time. From the clinical observation of muscular rigidity, many researchers have been interested in the stretch reflex response. They defined the M1 response as a tendon jerk and the M2 response as a long latency stretch reflex, and the M2 response had twice the tendon jerk latency with a much larger amplitude than the M1 response. Lee and Tatton examined the long latency stretch reflex in wrist flexor muscles of PD patients [3]. They observed an exaggerated M2 response, while the M1 response was unchanged. However, Rothwell et al. measured the long latency stretch reflex in triceps brachii and flexor pollicis longus muscles in PD patients with severe rigidity and showed that the M2 response was greatly increased in triceps brachii, whereas a normal M2 response was observed in flexor pollicis longus [4]. They concluded that enhanced long latency reflexes contribute to, but may not be solely responsible for, rigidity. Thus which components contribute to rigidity is still unclear. Activation rigidity, which is the clinically well-known phenomenon of reinforcing rigidity in one limb by requesting a voluntary flexion or extension in the other limb, is thought to indicate the central nervous system influence in the pathogenesis of rigidity [5]. Although activated rigidity may affect the long latency reflex system, it had not previously been adequately validated in clinical practice.

We previously succeeded in systematically analyzing factors of rigidity perceived by physicians in clinical examinations [6]. The results showed that the elastic coefficient (elasticity) and the difference in bias (difference in torque during flexion and extension) are factors in rigidity and that rigidity is perceived to be strong when either or both of these factors are large. We then considered the elastic coefficient, one of the component factors of rigidity, not as having one feature over the full joint angle range but as a model combining two elastic characteristics with different features. We previously showed the validity of the technique of analyzing elbow joint movement divided into angles proximal and distal to a joint angle of 60° [7]. In this study, the elbow joints of PD patients were moved passively at different velocities, and two components of rigidity were evaluated to determine whether they were velocity-dependent.

## 2. Methods

### 2.1. Subjects

This study included 20 patients (10 men and 10 women; mean age: 74.4 ± 6.2 years) diagnosed with PD according to British Brain Bank clinical criteria [8]. PD patients were assessed using UPDRS (Unified Parkinson Disease Rating Scale) Part III, and rigidity was scored using a five-point scale (0 = no rigidity, 1 = slight, 2 = mild to moderate, 3 = marked, and 4 = severe). The rigidity detected only during activation was not rated as score 1 or 0, because the patients were instructed to remain relaxed during the measurement and no movement was induced. The upper limb of the side that showed more severe rigidity was analyzed in each subject; it was the left side in 16 patients and the right side in 4 patients. In the present study, the UPDRS rigidity score was 1 in 8 patients, 2 in 9 patients, and 3 in 3 patients. All patients were on medication during the UPDRS assessment and during the measurements. All of the present subjects underwent head MRI, but no central nervous system lesions that would cause spasticity in the arms were seen. This study was approved by the Institutional Review Board of Toneyama National Hospital, and written, informed consent was obtained from all subjects in accordance with the Declaration of Helsinki.

### 2.2. Muscle Tone Measurement Device and Protocols

**Figure 1:**
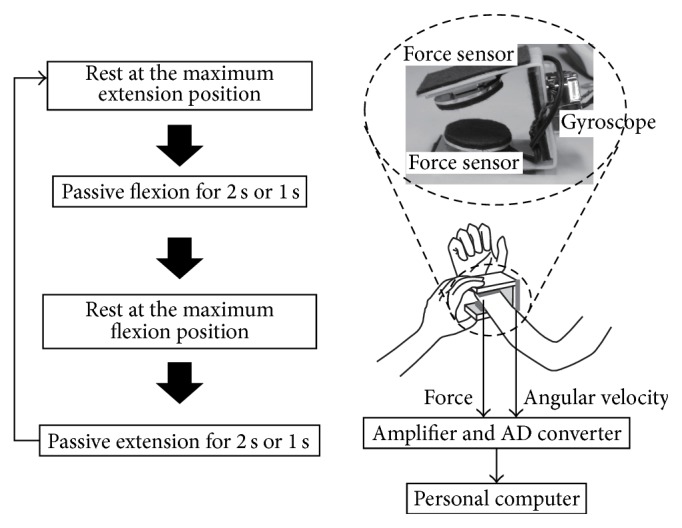
Overview of the muscle tone measurement system and the measurement protocol.

Figure 1 shows an overview of the muscle tone measurement system and the measurement protocol. This device consisted of small 3-axis force sensors and a gyro sensor. Two force sensors with soft pads were placed on either side of the wrist joint to measure the force perpendicular to the long axis of the arm during flexion and extension movements of the elbow joint and to calculate the torque at the elbow joint. The signals from the gyro sensor attached between the force sensors were used to calculate the angle of the elbow joint. The subjects were instructed to remain relaxed in the sitting position. An examiner held the elbow joint of the subject with one hand and the wrist joint of the subject with the other hand and performed passive flexion and extension of the elbow joint of the subject. The measurement was started from the maximum extension position. The following movements were repeated over 60 sec: more than 3 sec rest, flexion over 2 sec, more than 3 sec rest at the maximum flexion position, extension over 2 sec, and more than 3 sec rest at the maximum extension position. Each trial included five cycles of flexion and extension, and the times for flexion and extension were one time each for 2 sec (60°/s) and 1 sec (120°/s).

### 2.3. Data Analysis

**Figure 2:**
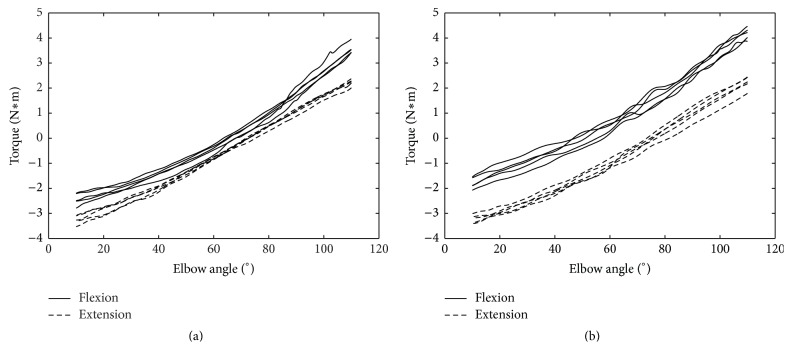
Angle-torque characteristics in passive flexion (solid line) and passive extension (dashed line) of left upper limb in one PD patient (UPDRS rigidity score = 3). The data included five cycles in (a) angular velocity 60°/s and (b) angular velocity 120°/s.

(1) Two different characteristic values, the elastic coefficient and the difference in bias, were extracted from the angle-torque characteristic plots during elbow flexion and extension in 20 PD patients. The angle-torque characteristic plots of one PD patient (UPDRS rigidity score = 3) were shown in Figure 2.
(2) Elastic coefficient: the data for joint angles of 10–110° were taken from the graphs of angle-torque characteristics of the elbow because inertial force may affect the data in the beginning and ending of the flexion and extension phase. The elastic coefficient was calculated by obtaining the slopes of the respective regression lines for flexion and extension.
(3) Difference in bias: bias was first defined as the torque value during flexion at one joint angle. It was defined similarly during extension. The difference in bias during flexion and extension was then calculated for the three angles of 30°, 60°, and 90°, and these values were summed.
(4) Statistical analysis: the elastic coefficients and the difference in bias, which were the characteristic values of muscle tone for the 20 PD patients, were analyzed statistically to determine whether there was velocity dependence in flexion and extension (JMP 11, SAS Institute Inc., Cary, NC, USA). Ten measurements, including five repetitive tests at two velocities, 60°/s and 120°/s, were recorded for each subject. Mixed-design analysis of variance for repeated measures was used for comparisons between the measurements of two velocities. Multivariate *F* tests were used because the sphericity chi-square test was significant.

## 3. Results and Discussion

**Figure 3:**
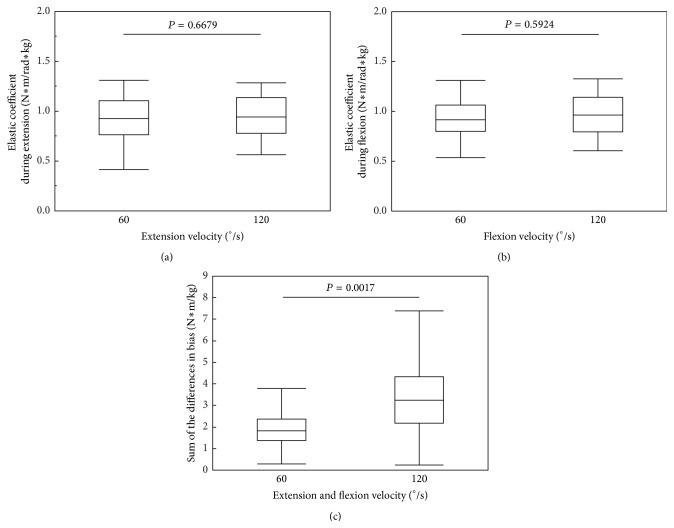
Changes in the (a) elastic coefficient during elbow extension, (b) elastic coefficient during elbow flexion, and (c) sum of the difference in bias with changes in extension and flexion velocity.

As shown in Figure 3, there was no difference in the elastic coefficient between the two velocities of 60°/s and 120°/s during extension (*P* = 0.6679) and flexion (*P* = 0.5924). In contrast, a significant velocity dependence was seen in a comparison of the sum of the differences in bias at 60°/s with that at 120°/s (*P* = 0.0017). The sum of the differences in bias increased as the velocity increased. The elastic coefficient and the difference in bias are two components of Parkinsonian rigidity, but they might have different features; the elastic coefficient has positional dependence as it is defined, and the difference in bias has velocity dependence.

The present result is the first to show velocity dependence in one component of rigidity that corresponds to clinical assessment.

Shimazu et al. observed the discharge patterns of the same single neuromuscular unit of the biceps brachii muscle with Parkinsonian rigidity on electromyography [9]. Their results suggest that a reduced spike-to-spike interval in rigidity tends to increase in the course of pallidotomy. It is possible that the decreased spike-to-spike interval in rigidity occurred because of not only overactivation of tonic motor units, but also a change to tonic in the phasic motor units as a result of excessive activity of afferent fibers from the muscle spindle. This interpretation is based on the report of Granit et al., who demonstrated that phasic alpha motor neurons show an increased stretch reflex and tonic properties during periods of excessive muscle spindle activity with the injection of succinylcholine [10].

Rothwell et al. demonstrated that the long latency stretch reflex in triceps brachii in PD patients with severe rigidity showed a larger response than normal controls, whereas a normal response was observed in flexor pollicis longus [4]. They also showed that the M2 response in flexor pollicis longus in PD patients with severe rigidity increased with velocity, although saturation occurred at velocities greater than 300°/s. In clinical examinations, rigidity is detected by passive movement in the main joint, and the physician cannot move the joint at a high speed over 300°/s. Moreover, it is hard for the examiner to assess the rigidity in flexor pollicis longus in such a small joint. The velocity-dependent component of Parkinsonian rigidity in the elbow joint may be derived from an exaggerated long latency stretch reflex.

Using this measurement system, we previously reported that Parkinsonian rigidity varies with joint angle [7]. Those findings, together with the present results, suggest that the features of rigidity may not conform to the conventional definition that rigidity is constant regardless of joint angle and does not depend on the speed at which the joint is moved. Differences in bias are high in subjects with a UPDRS rigidity score of 2 or greater. It may be said that physicians can feel velocity-dependency in some patients with moderate to severe rigidity because they evaluate rigidity as a combination of two components: the elastic coefficient and differences in bias.

Detailed analysis of spasticity using this technique may open the way to development of a unified model of muscle tone abnormality in neurological diseases.
